# Near-drowning-associated pneumonia with bacteremia caused by coinfection with methicillin-susceptible *Staphylococcus aureus* and *Edwardsiella tarda* in a healthy white man: a case report

**DOI:** 10.1186/s13256-016-0975-7

**Published:** 2016-07-16

**Authors:** Lucas Santos Zambon, Guilherme Nader Marta, Natan Chehter, Luis Guilherme Del Nero, Marina Costa Cavallaro

**Affiliations:** Emergency Department, Hospital das Clínicas da Faculdade de Medicina da Universidade de São Paulo, Av. Dr. Enéas de Carvalho Aguiar, 255, 5o andar, Sala 5023, Cerqueira Cesar, ZIP Code 05403-010 São Paulo, SP Brazil

**Keywords:** Near drowning, *Edwardsiella tarda*, Pneumonia, Bacterial, Bacteremia

## Abstract

**Background:**

*Edwardsiella tarda* is an Enterobacteriaceae found in aquatic environments. Extraintestinal infections caused by *Edwardsiella tarda* in humans are rare and occur in the presence of some risk factors. As far as we know, this is the first case of near-drowning-associated pneumonia with bacteremia caused by coinfection with methicillin-susceptible *Staphylococcus aureus* and *Edwardsiella tarda* in a healthy patient.

**Case presentation:**

A 27-year-old previously healthy white man had an episode of fresh water drowning after acute alcohol consumption. *Edwardsiella tarda* and methicillin-sensitive *Staphylococcus aureus* were isolated in both tracheal aspirate cultures and blood cultures.

**Conclusion:**

This case shows that *Edwardsiella tarda* is an important pathogen in near drowning even in healthy individuals, and not only in the presence of risk factors, as previously known.

## Background

The World Health Organization defines drowning as “the process of experiencing respiratory impairment from submersion/immersion in liquid” [1] emphasizing the importance of respiratory system damage in drowning pathophysiology, complications, and prognosis. More than 500,000 people die each year due to unintentional drowning [2]. According to the Center for Diseases Control, drowning was the tenth major cause of death related to injuries in the USA from 1999 to 2010 [3]. Approximately 50 % of drowning victims are under 20-years old [4]. In developing countries this incidence is even greater [5].

Lung infections are one of the most serious complications occurring in victims of drowning [6]. They may represent a diagnostic challenge as the presence of water in the lungs hinders the interpretation of radiographic images [5]. Both fungi and bacteria have been reported as etiological agents of after-drowning pulmonary infections [6]. Aerobic Gram-negative bacteria are the most frequently implicated in these infections [6].

*Edwardsiella tarda* is a facultative anaerobic flagellated Gram-negative bacilli member of the Enterobacteriaceae family found in aquatic environments [7]. This bacteria causes gastroenteritis predominantly. The main risk factors for extraintestinal infections are hepatobiliary diseases, iron overload syndromes, cancer, immunosuppression, and diabetes mellitus [8, 9].

As far as we know, the case about to be presented is the first documented episode of near-drowning-associated pneumonia with bacteremia caused by coinfection with methicillin-susceptible *Staphylococcus aureus* and *E. tarda* in a healthy patient. These data could motivate a different approach to antibiotic use for sepsis related to a near-drowning episode.

## Case presentation

A 27-year-old previously healthy white man had an episode of fresh water drowning after acute alcohol consumption. Friends quickly removed him from the water. A rescue team was activated and identified cardiopulmonary arrest in a non-shockable rhythm. Oral intubation was quickly performed. Neither stool reflux/vomiting nor aspiration was reported by the team. After two cycles of cardiopulmonary resuscitation (for about 4 minutes) and orotracheal intubation, return of spontaneous circulation occurred. During transportation bradycardia was reported, which reverted after one dose of atropine.

He was admitted to the emergency room of a tertiary academic hospital. On examination he was hemodynamically stable and comatose with 3 points on Glasgow Coma Scale (GCS) and nonreactive pupils. No other relevant physical findings upon arrival. He was placed on mechanical ventilation and transferred to the intensive care unit (ICU).

A few hours after admission to the ICU he presented decreased consciousness level (GCS 4), hypotension, and signs of poor peripheral perfusion. A blood gas analysis showed hypoxemia with respiratory acidosis. He underwent hypothermia for neuroprotection after cardiac arrest, received protective ventilation for acute respiratory distress syndrome (ARDS), and vasoactive drugs (norepinephrine plus epinephrine, which were maintained for 24 hours) through right subclavian central venous catheter (postpuncture pneumothorax was drained with a pigtail catheter uneventfully). He developed acute renal failure due to rhabdomyolysis, renal ischemia, and multiple organ failure, requiring hemodialysis for 15 days.

**Fig. 1:**
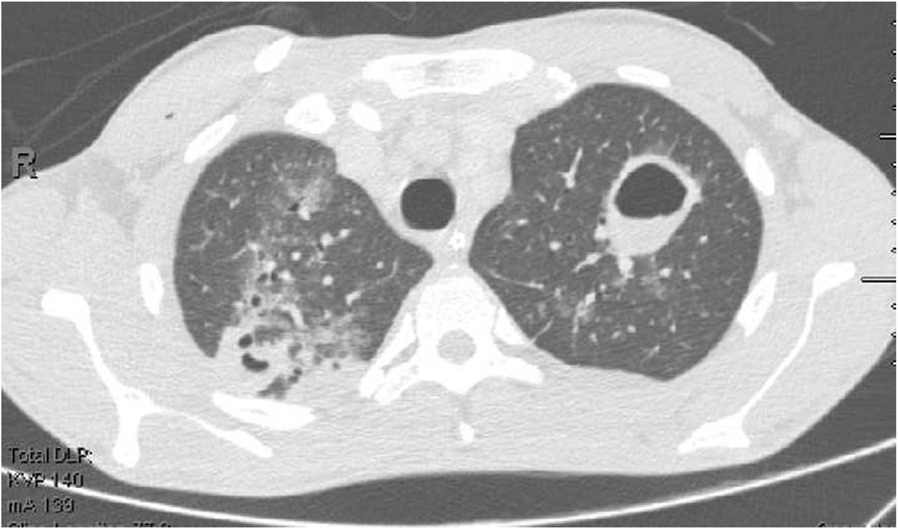
Upper third of the chest in computed tomography scan performed on the 11th day of hospitalization showed foci of pulmonary consolidation and cavities with thickened walls and fluid levels

**Fig. 2:**
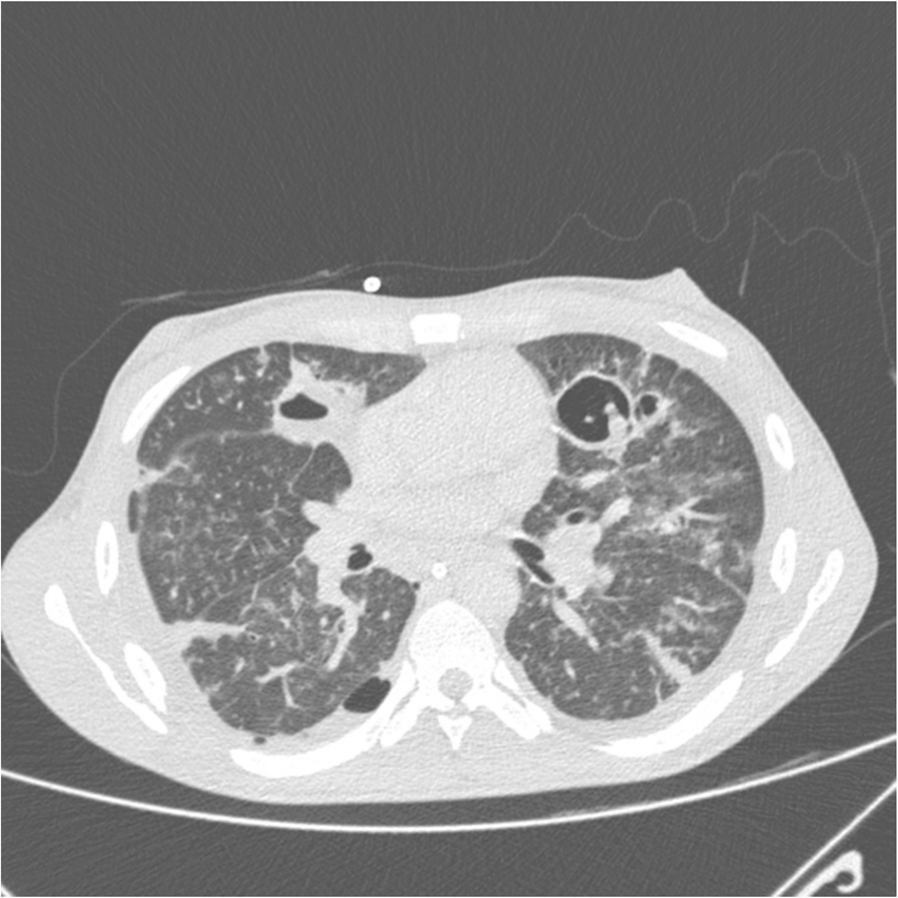
Middle third of the chest in computed tomography scan performed on the 11th day of hospitalization showed foci of pulmonary consolidation and cavities with thickened walls and fluid levels

Gram’s staining of his tracheal aspirate taken 3 days after the accident showed Gram-positive cocci isolated and in pairs, and frequent Gram-negative bacilli. Tracheal aspirate cultures isolated methicillin-sensitive *S. aureus*, *Enterobacter aerogenes*, *Aeromonas* species, and *E. tarda*. Blood cultures (first set obtained) isolated methicillin-sensitive *S. aureus* and *E. tarda*, which led to the introduction of oxacillin and ceftriaxone on the sixth day of hospitalization. Five more sets of blood culture were performed after the introduction of the antibiotics. All were negative. Computed tomography performed on the 11th day of hospitalization showed bilateral pleural effusion, and multiple pulmonary consolidations and cavities with thickened walls and air-fluid levels, consistent with lung abscesses (Figs. 1 and 2).

Twenty days after the ICU admission, he was transferred to the regular infirmary ward where the ongoing clinical, laboratory, and radiological improvement continued. On the 45th day of hospitalization, he was discharged home for out-patient monitoring with prescription of ciprofloxacin and clindamycin to be taken orally. He returned for follow-up consultation 14 days after taking the antibiotics. He reported no symptoms since the hospital discharge.

## Discussion

Although most victims of near drowning are previously healthy, the morbidity and mortality associated with these events are high, mainly due to pulmonary and neurological complications associated with tissue damage by hypoxia, acidosis, and hypoperfusion [6]. After submersion, the victim’s conscious response leads to a period of voluntary apnea, which stimulates the respiratory drive, leading to involuntary aspiration [5]. Aspirated water, in contact with the alveoli, leads to surfactant dysfunction and an increase in the alveolar–capillary membrane permeability, causing extensive pulmonary edema, atelectasis, and bronchospasm [10]. The combined effects of alveolar damage, contaminated material inoculation in the airways, and the frequent need of mechanical ventilation respiratory support result in an up to 12 % risk of after-drowning pneumonia [11]. This risk may vary according to the volume aspirated, the degree of water contamination and its temperature, as well as to the occurrence of aspiration of gastric content [6]. When admitted to an ICU, drowning victims should be managed following ARDS guidelines [5].

Lung infections are one of the most serious complications occurring in victims of drowning [6]. They may represent a diagnostic challenge as the presence of water in the lungs hinders the interpretation of radiographic images [5]. However, prophylactic antimicrobial therapy is not recommended due to the potential selection of resistant bacteria [12]. Both fungi and bacteria have been reported as etiological agents of after-drowning pulmonary infections [6]. Aerobic Gram-negative bacteria are the most frequently implicated bacteria in these infections, among which stand out *Aeromonas* species (in particular, *Aeromonas hydrophila*), *Burkholderia pseudomallei*, and *Chromobacterium violaceum* [6]. Gram-positive cocci such as *S. aureus* and *Streptococcus pneumoniae* and some Enterobacteriaceae are also reported as etiological agents of pneumonia, although it is often difficult to distinguish whether the infection was due to drowning or nosocomial related [6].

*E. tarda* is a facultative anaerobic flagellated Gram-negative bacilli member of the Enterobacteriaceae family found in aquatic environments [7]. Pathogenicity in humans, although rare, has been demonstrated predominantly in gastroenteritis, which represents more than 80 % of the infections by this agent [7–13]. Nonetheless, there are also reports of extraintestinal infections such as cellulitis and cutaneous abscesses, meningitis, endocarditis, osteomyelitis, liver abscess, tubo-ovarian and peritoneal abscess, as well as bacteremia and sepsis [8, 9, 11–14]. There is no report of pneumonia cases in immunocompetent patients so far. In the present case, only blood and tracheal aspirate cultures were performed. An endotoxin test was not available at the hospital. Nonetheless, the endotoxin is of secondary pathogenic importance when compared to infections caused by *Salmonella*, *Shigella* and *Yersinia* [15].

The most important risk factor for *E. tarda* infection is exposure to aquatic environments [13], and the main risk factors for extraintestinal infections are hepatobiliary diseases, iron overload syndromes, cancer, immunosuppression, and diabetes mellitus [8, 9].

## Conclusions

This is the first report of near-drowning-associated pneumonia with bacteremia by coinfection with methicillin-susceptible *S. aureus* and *E. tarda* in a patient without comorbidities, documented by isolation of the bacteria from blood cultures and in tracheal aspirate cultures. The only reported case of pneumonia caused by *E. tarda* (isolated only in sputum) occurred in a patient hospitalized for diabetic ketoacidosis, with no history of drowning [9]. There are no reports of pneumonia caused by *E. tarda* in a patient without previous medical history, nor reports of *E. tarda* bacteremia from pulmonary infection. The capacity of *E. tarda* to form abscesses in other parts of the body such as skin, ovaries, and liver has already been well documented [8, 9, 14]. This may suggest its involvement in the formation of extensive lung abscesses in this case in association with the *S. aureus*, although there are also no reports of such clinical presentation.

Although extraintestinal *E. tarda* infections are susceptible to most antibiotics that target Gram-negative bacteria, including β-lactams, aminoglycosides, quinolones, tetracyclines, and chloramphenicol [9, 13, 16], the lethality of extraintestinal *E. tarda* infections is approximately 23 % [8], reaching rates as high as 50 % in cases of bacteremia [13].

This case widens the spectrum of extraintestinal presentations of *E. tarda* infection to include bacteremia from lung infection. Thus, the monitoring of drowning victims for pulmonary infection should be thorough and should always include sputum cultures to allow detection of waterborne bacteria which, although rarely isolated, can cause highly lethal infections.

## Abbreviations

ARDS, acute respiratory distress syndrome; GCS, Glasgow Coma Scale; ICU, intensive care unit.
